# Mobile Self-Monitoring ECG Devices to Diagnose Arrhythmia that Coincide with Palpitations: A Scoping Review

**DOI:** 10.3390/healthcare7030096

**Published:** 2019-08-16

**Authors:** Hannah Ramsden Marston, Robin Hadley, Duncan Banks, María Del Carmen Miranda Duro

**Affiliations:** 1 Health and Wellbeing Priority Research Area, School of Health, Wellbeing and Social Care, Faculty of Wellbeing, Education and Language Studies, The Open University, Buckinghamshire, Milton Keynes MK7 6AA, UK; 2 School of Life, Health & Chemical Sciences, Faculty of Science, Technology, Engineering & Mathematics, The Open University, Milton Keynes MK7 6AA, UK; 3 Faculty of Health Sciences, Department of Physiotherapy, Medicine and Biomedical Sciences, Oza Campus, University of A Coruña, A Coruña 15006, Spain

**Keywords:** cardiology, wearable devices, community care, primary care, technology, clinical care, scoping review

## Abstract

The use and deployment of mobile devices across society is phenomenal with an increasing number of individuals using mobile devices to track their everyday health. However, there is a paucity of academic material examining this recent trend. Specifically, little is known about the use and deployment of mobile heart monitoring devices for measuring palpitations and arrhythmia. In this scoping literature review, we identify the contemporary evidence that reports the use of mobile heart monitoring to assess palpitations and arrhythmia across populations. The review was conducted between February and March 2018. Five electronic databases were searched: Association for Computing Machinery (ACM), CINHAL, Google Scholar, PubMed, and Scopus. A total of 981 records were identified and, following the inclusion and exclusion criteria, nine papers formed the final stage of the review. The results identified a total of six primary themes: purpose, environment, population, wearable devices, assessment, and study design. A further 24 secondary themes were identified across the primary themes. These included detection, cost effectiveness, recruitment, type of setting, type of assessment, and commercial or purpose-built mobile device. This scoping review highlights that further work is required to understand the impact of mobile heart monitoring devices on how arrhythmias and palpitations are assessed and measured across all populations and ages of society. A positive trend revealed by this review demonstrates how mobile heart monitoring devices can support primary care providers to deliver high levels of care at a low cost to the service provider. This has several benefits: alleviation of patient anxiety, lowering the risk of morbidity and mortality, while progressively influencing national and international care pathway guidelines. Limitations of this work include the paucity of knowledge and insight from primary care providers and lack of qualitative material. We argue that future studies consider qualitative and mixed methods approaches to complement quantitative methodologies and to ensure all actors’ experiences are recorded.

## 1. Introduction

In the past two decades, there has been a phenomenal increase in the take-up of wearable mobile devices, with many facilitating the measurement of a variety of health outputs. While many of these devices are basic, there are a number of devices that offer clinician-level diagnostic evaluations. According to Public Health England (PHE), in the United Kingdom (UK), there are 1.4 million people or 2.5% of the population who have atrial fibrillation (AF) [1]. Given the rise of these estimated projections in addition to PHE purporting AF increases with age, in particular, with 80.5% of the English population aged 65 years and over experiencing AF and a further 985,000 people living in England, UK, with undiagnosed AF, equating to 425,000 people [1]. Moreover, there are global implications: the United Nations (UN) estimate that there will 8.6 billion people by 2030, increasing to 9.8 billion in 2050 [2]. Consequently, there is an opportunity to determine whether mobile devices can provide a timely and cost-effective solution to identify the risk of AF.

The purpose of this review is to explore the current trends in the use of wearable stand-alone devices capable of recording the electrical activity of the heart electrocardiogram (ECG/EKG) used for the detection of cardiac arrhythmias associated with palpitations.

People describe palpitations as a feeling that their heart is pounding or fluttering or that their heartbeat is irregular [3,4]. These feelings can last from a few seconds to several minutes and patients often perceive them as a serious cause for concern [3,4]. There are many reasons for palpitations including changes in emotional or psychological state, the use of hormones, prescribed and illegal drugs, excessive alcohol, smoking, strenuous exercise, and excessive consumption of caffeinated drinks. In most cases, palpitations invariably raise a person’s anxiety, leading to increased visits to their General Practitioner (GP) or hospital. Furthermore, it has been established that palpitations are connected to greater morbidity, a higher risk of stroke, heart failure and an increase in risk of mortality [5,6,7,8,9]. In the UK, the internationally recognised organisation “The Arrhythmia Alliance note four out of 100 people aged ≥65 years are affected by AF one of the most common types of arrhythmia (AR)” [3]. Patients present with varying symptoms including palpitations, shortness of breath or chest pains. However, some people may not display any symptoms, but other indications will lead to detection [3]. Repeated visits to the GP lead to the phenomenon of the ‘worried well’: patients who may feel they are wasting health practitioners’ time and adding unnecessary costs on to the health service. However, people’s quality of life (QOL) is severely affected by this health complaint. If AR is suspected, the current recommended advice is to monitor a patient either in a hospital environment or to wear a 24-h ECG device such as Holter monitor [3,4]. The recent development of substantially cheaper wearable technologies provides a challenging alternative to the traditional approach.

Given the rise in ageing populations, a reduction in health care services and additional strains on the delivery of primary care, there is a greater need to explore alternative, accurate and cost-effective solutions to detect and diagnose AF. Mobile ECG devices are worth considering because they can reduce the diagnosis time and have the potential to be cost effective, while enabling heart activity to be monitored over a prolonged period [10]. By contrast, the traditional alternatives are uncomfortable to use and can only be worn for a very limited amount of time or, in the case of implanted loop recorders, they require invasive surgery [11]. Moreover, since 2010, wearable devices such as Fitbit devices, Jawbone UP, Garmin Vivofit and Misfit Shine have increasingly been used to monitor and analyse one’s daily activity through self-tracking users’ progress over time. Usually, goal-oriented [12] tasks over a set period are agreed by the user (i.e., walk 10,000 steps per day). In addition, these devices often offer rudimentary Heart Rate (monitoring). The user can then review their progress, share their data with their friends, family and health practitioner (i.e., physician, nurse or consultant). Contemporary evidence provides an insight into the use of mobile heart monitoring via wearable devices to measure heart rate and rhythm [9,13,14,15]. Cheung, Krahn, and Andrade [9] discuss the current and ongoing developments of wearable devices, which have entered the consumer market at a phenomenal rate. Consequently, physiological data, sleep patterns, Heart Rate (HR) and much more have been tracked. Cheung, Krahn and Andrade described the various wearable devices that have the ability to track HR and AR [9]. Nonetheless, Cheung, Krahn and Andrade note that one of the limitations of wearable devices is the level of accuracy, which has only been evaluated on small sample sizes of patients presenting with unique symptoms [9]. Furthermore, Cheung, Krahn and Andrade argue that the suitability of wearable devices for detecting or for the treatment of AF within clinical settings remains unanswered [9].

### 1.1. Overview of Mobile ECG Devices

There are several mobile ECG devices available on the market. Firstly, the HeartCheck™ ECG Pen [16] has received Food and Drug Administration (FDA) approval for monitoring AR. The user/patient does not require a prescription to access/use the device and has Internet access to a qualified physician. The HeartCheck™ device is easily transportable given the size of the device—similar to a pen—and enables the user/patient to take their reading(s) anywhere [15]. Quinn et al. [17] conducted a clinical trial with the HeartCheck™ ECG Pen involving 22 primary care clinics, 2054 participants aged 65 years and older (mean age = 73.7 ± 6.9). Participants had to be attending clinics/appointments on a regular basis and were required to undertake three different types of screening methods in the trial. The first screening method was a 30-s radial pulse check and the second a single-lead ECG. The third consisted of a screening blood pressure machine with AF detection. Participants who presented positively with one or more tests were then required to undergo a 12-lead ECG with or without 24-h Holter. Participants with confirmed AF received a 90-day follow up. The overall findings [16] showed the single-lead ECG and the blood pressure devices to have superior specificity in comparison to the pulse check. Fifty-six (2.7%) participants were confirmed with AF: 12 newly diagnosed and 44 previously diagnosed.

The EMAY mobile ECG device [18] is available to purchase from Amazon for £79.00. The company states the device is ‘intended for initial evaluation’ and ‘not for medical diagnostic use’ [18]. The EMAY device is used by both hands when taking a reading and can be used anytime and anywhere. The EMAY website notes patients with a myriad of health conditions such as chronic disease, coronary heart disease, diabetes, hypertension, myocarditis, obesity, chest pain, palpitations and dyspnea can use the device [18]. Additional information on the EMAY website states several ‘cardiac situations that could be detected’ that include missed beat, tachycardia, bradycardia, arrhythmia, Accidental Ventricular Premature Beats (VPBs), VPB trigeminy, VPB bigeminy, VPB couple, VPB runs of 3, VPB runs of 4, VPB RonT, ST elevation, and ST depression [18]. To date the clinical trials website returned no registered clinical trials using the EMAY mobile ECG device [19].

The Beurer ME 90 Bluetooth^®^ mobile ECG device purports to accurately monitor and record users/patients heart rhythm on the go, or at home. This device is compatible with iOS 8.0, Android 4.4, Bluetooth 4.0 or above platforms. The device is categorised as a medical device and users/patients have the ability to transfer recordings over Bluetooth. The Beurer ME 90 Bluetooth^®^ has a USB portal and has storage for 36 recordings. The device is CE marked and is covered by German health insurance and pharmaceutical legislation [20]. To date, the clinical trials website returned no registered clinical trials using the Beurer ME 90 Bluetooth^®^. The device is available to purchase from Amazon DE for €137.72 or through a third party for approximately £134.55.

The AliveCor Kardia Mobile ECG device is available to purchase directly from the company website or via other third-party websites for £99.00. The AliveCor device is available on both Android and iOS platforms and it is noted the device should not be used with pacemakers or ICDs [21]. AliveCor state that the device can detect AF instantly and is CE marked with positive National Institute for Health Care Excellence (NICE) advice. AliveCor Kardia [21] declare the device has been clinically proven and is used by leading cardiologists. Users/patients can track their weight and blood pressure within one app and has the option to take unlimited EKG recordings. Users can take a recording in 30 s using their thumbs pressing down on the pads. Users have additional options to pay for a premium membership, which enables them to receive unlimited history and storage of EKG, and monthly reports. To date, the company website reports a total of 69 peer-reviewed articles [22] using the AliveCor mobile ECG device within a myriad of varying health cohorts and chronic diseases.

### 1.2. Background Literature

Limited studies have examined how wearable devices perform compared to the Holter ECG. For example, Pevnick et al.’s [23] retrospective paper explores existing wearable devices, which have been designed specifically to measure activity, heart rate (HR) and heart rhythm. However, this paper provides limited information and lacks critical insight into the deployment of mobile ECG monitoring in primary care settings. Furthermore, it does not account for the perspective of health practitioners, physicians, and cardiologists. Likewise, their proposed frameworks and taxonomies lack clarity and theoretical underpinning, resulting in a paucity of in-depth knowledge and experience of these devices. In the UK, the National Institute for Health Care Excellence (NICE) [10] provides health information guidance, policy and practice, procedures, and standards. This guidance is informed on evidenced-based studies for clinical practitioners, public health practitioners, and social care institutions employed across the National Health Service (NHS). In 2015, The Newcastle and York External Assessment Centre and the Medical Technologies Evaluation Programme, NICE [10], conducted a literature search to identify evidence-based research and the cost effectiveness of the AliveCor ECG device and the AliveECG App. A total of eight databases were searched, resulting in 1033 records retrieved. After screening, four papers were identified that met the review’s inclusion criteria: Lau et al. [24], Lowres et al. [25], Haberman et al. [26] and Tarakji et al. [27]. The review acknowledged that there were other mobile ECG devices available. For example, Dicare m1CC Colour portable ECG recorder (Dimetek), MD100A ECG reader (Choice Medical), MD100E ECG reader (Choice Medical), and HCG-801 ECG reader (Omron). Moreover, the review reported further information from ‘Specialist commentator comments’ and the ‘Patient and carer perspective’ of The Arrhythmia Alliance and the Atrial Fibrillation Association, respectively. While the commentary in the briefing can be taken positively; it is unclear why the review focused specifically on the AliveCor ECG device and the AliveECG app [22].

The Zenicor mobile ECG device was developed by the Swedish-based Zenicor Medical Systems AB. [28]. The Zenicor ECG device enables readings to be taken by the user/patient by placing their thumbs on two electrodes for 30 s. This device supports a web-based service that enables the analysis, interpretation, presentation, and processing and storage of the ECG recordings, to the care provider. The Zenicor ECG device is CE marked and is ISO (International Organization for Standardization) 13485 rated [29]. The Zenicor Medical Systems AB website lists three pieces of evidence-based research (published in English) of the Zenicor mobile ECG device. These studies were conducted by Hendrikx et al. [13], Usadel et al. [14], and Dahlqvist et al. [15]. Each used the Zenicor ECG mobile device to explore AR. Both Hendrikx et al. [13], and Dahlqvist et al. [15] recruited participants aged 18≥ years. Usadel et al. [14] focused on AR in children aged between 5 and 17 years. Hendrikx et al. [13] conducted a prospective, observation, cross-sectional study within a hospital’s clinical physiology department. Hendrikx et al. [13] recruited 108 participants, who had been referred to clinicians for ambiguous palpitations, or experiences of dizziness. In total, 95 patients (42 men and 53 women) were assessed with a mean age of 54.1 years. All the participants were given a 24-h Holter ECG in addition to the Zenicor EKG handheld (for 30 s). Readings were taken twice a day when the participants were experiencing symptoms. The results from the 24-h Holter ECG ascertained two patients with AF and a third with atrioventricular (AV), a further three patients displayed paroxysmal supraventricular tachycardia (PSVT), and another patient presented with AV-block-II. Hendrikx et al. [13] concluded the use and deployment of the Zenicor EKG handheld to be more effective than the 24-h Holter ECG in detecting AF and PSVT, specifically with patients experiencing ambiguous symptoms.

The study by Usadel et al. [14] examined patients aged 0–17 years, who have or did not have congenital heart defects, pacemaker/ICD or AF and compared a lead-12 ECG with the Zenicor EKG handheld. Recordings and the transmission of data were completed successfully by the Zenicor EKG device with thorough and consistent data readings. The P wave detection was reported to be challenging, with 82 participants displaying heart rhythm disturbances. The detection of sensitivity via the Zenicor EKG handheld identified 92% of participants diagnosed with supraventricular tachycardia, while abnormal ECGs were identified with 77 and 92% sensitivity and specificity, respectively. In conclusion, Usadel et al. [14] noted that the use of the Zenicor EKG device with children was appropriate. Moreover, they suggested that the device was a suitable tool for detecting and excluding tachycardia in children. Dahlqvist et al. [15] evaluated the Zenicor EKG handheld to ascertain whether AF and cardiac autonomic dysfunction can be diagnosed in children with univentricular hearts. A total of 27 patients were recruited and used the Zenicor EKG handheld over a period of 14 days, while a manual AF analysis was conducted. The results from this study identified asymptomatic AF in one patient while HRV was also identified in some patients. Dahlqvist et al. [15] concluded that the use of the Zenicor EKG handheld device was a useful tool for detecting AF and cardiac autonomic dysfunction.

Reed et al. conducted a randomised control trial (RCT) [30] across multiple sites in the UK, deploying the AliveCor ECG device to ascertain symptomatic rhythm detection in patients attending the emergency department. Reed et al. [30] recruited 242 participants over a period of 18 months, with 125 allocated to the intervention group and 117 to the control group. The findings from the RCT were positive and the primary outcome of identifying symptomatic rhythm detection was identified in 69 participants in the intervention group, and 11 participants in the control group. The length of time to identify the primary outcome was 9.5 days for those participants assigned to the intervention group and 42.9 days for those participants assigned to the control group. Symptomatic cardia was detected in 11 intervention group participants and in one person in the control group. During the RCT phase, a total of seven questions were posed to participants assigned to the intervention group to ascertain patient engagement and usability of the AliveCor ECG device. Overall, the results showed positive responses to engaging and using the ECG device. The majority of participants (70%) reported to have never using a mobile ECG device; 21.6% of participants strongly agreed that the AliveCor ECG device will be useful in diagnosing their symptoms; 22.4% reported positively to recording their heart tracing based on their initial experiences upon entering the emergency department. Furthermore, 28.0% of participants reported having no problems or concerns when they sent a heart trace to the study team, while 32.0% of participants strongly agreed with having no problems recording a trace. Reed et al. [30] reported that their study demonstrated the cost benefits of using the AliveCor ECG device relating to primary, community and secondary care for both the intervention and control groups. The authors identified a £108 cost saving for participants in the intervention group. While no cost saving was identified for the control group, further analysis found that the cost saving per symptomatic rhythm diagnosis was less per patient in the intervention group (£474) compared to the control group (£1395). Reed et al. [30] argue that their findings are generalizable from emergency medicine to general practice, across a myriad of health care systems. However, Reed et al. [30] study reported findings based on the use and deployment of one particular ECG device. While they did not state the justification(s) for choosing the AliveCor ECG device over other devices available on the market, the results have demonstrated the positive effects of using a mobile ECG device.

Acknowledging the growing popularity of mobile heart monitoring (including ECG) devices, a search of the clinicaltrials.gov website [31] was undertaken. The website displays information concerning clinical trials that are either completed, active, recruiting, not recruiting or unknown. Three individual searches were conducted using the search terms ‘Alivecor [32] ECG device’, ‘Zenicor ECG device’ and ‘atrial fibrillation and wearable devices’. The device terms were used because of the studies referred to earlier. The latter term was used to capture any other device(s). Regarding the Alivecor [32] ECG device, 25 registered trials between the years 2013 and 2019 were identified. The majority of the 25 studies were conducted in the USA (n = 17). The remainder were conducted in Canada (n = 2), Hong Kong (n = 2), UK (n = 1), Belgium (n = 1), India (n = 1), and the Netherlands (n = 1). Five trials between 2012 and 2019 were identified involving the Zenicor ECG device [33]. The majority had been conducted in Sweden (n = 3), but also included one in Germany (n = 1) and one conducted in multiple locations (Denmark, Sweden and Austria). The final search using the terms ‘atrial fibrillation and wearable devices’ (e.g., Garmin Smart Watch, Amiigo Watch and Wristband, and iRhythm Zio XT Patch) yielded a further 17 trials between 2012 and 2019 [34]. Five trials were conducted in the USA (n = 5), two in Canada (n = 2), two in Finland (n = 2), two in Israel (n = 2), and one each in Belgium (n = 1), China (n = 1), Germany/Switzerland (n = 1) Singapore (n = 1), Spain (n = 1) and in the UK (n = 1), respectively. The HeartCheck™ device was used in one RCT study conducted in Canada [35].

Although this complex and rapidly changing field represents an attractive prospect for the diagnosis of heart and circulatory disease, there are few reviews that look at mobile self-monitoring ECG devices designed to diagnose cardiac arrhythmia that coincide with cardiac event-related conditions such as palpitations. This paper reviews the contemporary literature and examines whether the evidence obtained from studies with such devices can support primary care providers to deliver high levels of care at a low cost to the service provider. Although the causes of palpitations are variable, they are occasionally a manifestation of potentially life-threatening arrhythmia (AR). Under conditions where abnormal heart rhythms and cardiac symptoms are irregular and infrequent, such mobile self-monitoring ECG devices have potential as event monitors during symptoms such as palpitations. Cheung, Krahn, and Andrade [9] argued that one of the limitations of wearable device studies was their reliance on small sample sizes involving patients presenting with unique symptoms.

This review is distinctive and timely in that it provides an insight into an increasingly complex field that combines the precision demanded by the medical profession with wearable technology that has advanced rapidly with the development of miniaturised, and increasingly accurate, sensors. The authors believe it is the first review of its kind to explore contemporary evidence surrounding the use of mobile ECG devices and, consequently, contributes to the field of primary care and medicine. The authors aim to offer further evidence for the support of such devices in a community setting and to answer the question of what contemporary evidence reports the use of mobile ECG monitoring to assess palpitations that occur with AR across populations.

## 2. Methods

A scoping review strategy was selected to chart this important and complex subject. Arksey and O’Malley [36] propose that scoping reviews provide a clear and thorough method for providing an overview of significant and quickly developing areas of research. Furthermore, Arksey and O’Malley [36] note the aim of this type of review is ‘to illustrate the field of interest in terms of the volume, nature and characteristics of the primary research’ (p. 30).

In order to chart the emerging nature of the rapidly developing area of mobile heart measuring devices, a scoping review was deployed using Arksey and O’Malley’s [36] framework. This framework gives both an overview of the topic and facilitates an examination of the breadth and depth of knowledge of the subject. One of the framework’s strengths is that it allows the authors to draw conclusions about the overall state of research activity and make recommendations for future research.

### 2.1. Objectives

This review was guided by Arksey and O’Malley’s five-stage framework [36], which includes (a) establishing the research question, (b) the identification of pertinent studies, (c) the choice of studies, (d) mapping the data and (e) collating, summarizing and reporting the findings.

### 2.2. Search Strategy

The search strategy consisted of a systematic search of five electronic databases. The databases examined were the Association for Computing Machinery (ACM, New York, NY, USA), CINHAL, Google Scholar, PubMed, and Scopus. The date criterion of the search was for material published between January 2010 and February 2018. The search was conducted between February and March 2018.

Each database underwent individual search strategies and the limiters were ‘English’ and ‘humans’. Articles, their references (BibTeX format) and, where possible, the CSV files were exported into Dropbox and Mendeley. An inclusion and exclusion criteria were developed and deployed. The complete search criteria are given in Table 1.

### 2.3. Selection Criteria

Studies were included if they met the inclusion criteria (Table 1). Titles of papers and abstracts were initially screened for suitability and, where necessary, the full paper was then reviewed. The final decision was determined by two authors (H.M. and D.B.). Both H.M. and D.B. reviewed all articles from each database separately and then collectively. Where additional discrepancies were highlighted, H.M. and D.B. reviewed and discussed the respective paper(s) before a final decision was made. Both abstracts and full texts were retrieved to determine whether they met the inclusion/exclusion criteria. Both H.M. and D.B. jointly decided the final selection of papers for inclusion.

## 3. Results

**Figure 1:**
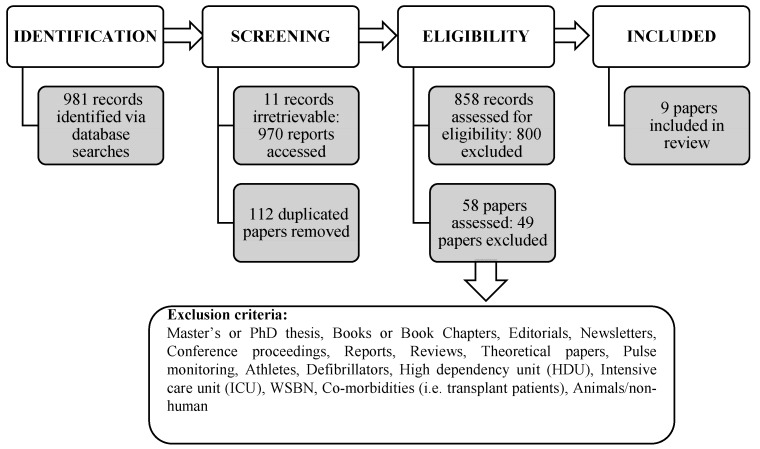
Diagram showing the review process.

The initial search yielded 981 records across the five databases. However, 11 records were not available (six from CINHAL and five from Google Scholar). Consequently, 970 records were accessed, and 112 duplicates removed. The remaining 858 records were then judged against the inclusion/exclusion criteria (see Table 1). Subsequently, 800 records failed the inclusion criteria and were excluded from the review. The remaining 58 papers were subjected to a full text assessment and a further 49 papers were excluded (Figure 1). Nine papers met the inclusion criteria of the review.

### 3.1. General Characteristics of Studies

Analysis of the final nine articles found that they were all published between 2015 and 2017 and the majority were located in the PubMed database (n = 5). The sample size varied across all articles from 25,415 participants in the study published by Arronsson et al. [37] to 22 participants in the study published by Doliwa et al. [38]. The total sample includes 27,346 participants with a mean age of 57.18 ± 7.42, and the median age is 64.45. Five studies reported the percentage or number of female participants [37,38,39,40,41,42], while three studies recruited only male participants [26,43,44]. Three studies were performed in Europe: Arronsson et al. [37], Doliwa, Rosenqvist and Frykman [38], and Halcox et al. [42]. Five studies were conducted in the USA: Boudreaux et al. [39], Turakhia et al. [44], Haberman et al. [26], Hickey and Freedson [41] and McManus et al. [43]. The remaining study was conducted in Africa by Evans et al. [40]. Study design varied, with three studies reporting an observation cohort study Arronsson et al. [37], Halcox et al. [42], Haberman et al. [26]. Two studies reported on an experimental study approach: Evans et al. [40] and McManus et al. [43]. Hickey and Freedson [41] reported an experimental comparative study design. A further two studies reported a prospective observation study design: Boudreaux et al. [39] and Halcox et al. [42]. One study, Doliwa, Rosenqvist and Frykman, reported an experimental RCT design [38].

### 3.2. Themes

A total of six primary themes and the 24 secondary themes which were identified through the review process. The secondary themes detail the type of assessments used in the studies.

### 3.3. Primary Themes

A total of six primary themes (purpose and objectives, environment, population, wearable devices, assessment, and study design) were identified and are explained in the proceeding sections.

The purpose and objectives theme comprised five secondary themes: detection, feasibility, comparison, cost effectiveness and study protocol. The Halcox et al. [42] study does not directly report the purpose of the study. However, critical examination of the complete paper revealed, that Halcox et al. [42] compared the AliveCor device with the delivery of routine care [42].

Four studies, Arronsson et al. [37]; Boudreaux et al. [39]; Turakhia et al. [44] and McManus et al. [43] used the detection of AF to assess the ECG device. Evans et al. [40] explored the feasibility of the AliveCor ECG device. The study by Boudreaux et al. [39] compared the energy expenditure (EE) during a particular activity (i.e., resistance training). Haberman et al. [26], and Doliwa et al. [38] compared the AliveCor mobile ECG device against the traditional method of a 12-lead ECG. Cost effectiveness (CE) was also assessed by Arronsson et al. [37]. We have included the McManus et al. study protocol that outlines a single-centre, prospective randomised control trial (RCT) that deployed the AliveCor ECG device over a 30-day period [43]. This study protocol reported three aims: (a) to document AF using real-time ECG capture; (b) to evaluate the impact on AF treatment and Quality-Adjusted Life Years (QALYs—A generic measure of disease burden); and (c) to evaluate the effectiveness of text messaging on AF knowledge and proactive self-management of multiple chronic conditions.

The environment theme highlighted the different types of environment where studies were undertaken. Four secondary themes were identified: multiple screening centres, university/laboratory, rural community/hospital and a Veteran Affairs centre. The Aronsson et al. [37] study was located in multiple screening centres (n = 6) as reported in their earlier publication by Friberg et al. [45]. Five studies took place in university/laboratory settings Boudreaux et al. [39], Halcox et al. [42], Haberman et al. [26], McManus et al. [43], and Hickey et al. [41]. One study by Doliwa, Rosenqvist and Frykman was conducted in a hospital setting [38]. The study by Evans et al. [40] took place in a rural community/hospital where cardiology resources were limited. Moreover, there was only one 12-lead ECG tape available. The Turakhia et al. [44] study was conducted at the Veteran Affairs (VA) Palo Alto Health Care System.

The population theme encompasses three secondary themes: recruitment, sample, and sample size. Across all the studies, the nature of recruitment varied and included hospital clinics by Halcox et al. [42], the recruitment of veterans by Turakhia et al. [44], specific cardiology clinics/departments by Evans et al. [40], Haberman et al. [26], and McManus et al. [42], and university students by Haberman et al. [26]. The sample also varied and included older adults [44], athletes [26], healthy adults [26] and those with existing comorbidities (i.e., coronary disease, heart failure) [26]. The size of samples ranged from Aronsson et al. [37]’s 25,415 participants [37] to Doliwa, Rosenqvist, and Frykman’s 22 participants [38]. Boudreaux et al. [39] recruited participants aged between 18 and 35 years, while Aronsson et al. [37] primarily recruited adults aged between 75 and 76 years. The majority of studies recruited participants of both genders with the exception of Turakhia et al. [44] who recruited only male participants.

The wearable devices theme encompassed three secondary themes: commercial, wearable patch and purpose-built devices. Eight studies utilised commercial devices. Two studies used the Zenicor ECG device: Aronsson et al. [37], and Doliwa, Rosenqvist, and Frykman [38]. One study by Boudreaux et al. [39] used multiple heart rate measuring mobile devices (i.e., Apple Watch Series 2, Fitbit Blaze, Fitbit Charge 2, Polar H7, Polar A360, Garmin Vivosmart HR, TomTom Touch, and Bose SoundSport Pulse headphones). Five studies—Evans et al. [40] Halcox et al. [42] Haberman et al. [26], Hickey and Freedson, [41] and McManus et al. [43]—Used the AliveCor Kardia mobile device, which is attached to an Apple iPhone. One study, Turakhia et al. [44], deployed the Zio wearable patch that sits against chest skin. Finally, McManus et al. [43] used a purpose-built mobile app (mApp) called PULSE-SMART to undertake participants ECG readings and that was connected to an Apple iPhone 4S.

The assessment theme encapsulates 11 secondary themes: the completion of assessment (i.e., health practitioner); self-assessment surveys—non-validated (health—anxiety, perceived benefits from health care practitioners), qualitative data (i.e., patient diary of symptoms); AF scales for the Assessment, Medication Assessment, Other Assessment, Quality of Life, Anxiety Scale; Technology-based Assessment and ECG monitoring (Holter or Mobile device) and patient health medical records (demographics, medical history, health behaviours). Due to the number of themes, this section only states theme type and one respective study. The completion of assessment by a health practitioner: Evans et al. [40]. Self-assessment surveys—non validated (health—anxiety, perceived benefits from health care practitioners): Doliwa et al. [38]. Qualitative data (i.e., patient diary of symptoms): Turakhia et al. [44]. AF scales for Assessment: Hickey et al. [41]. Medication Assessment, Other Assessment: Hickey et al. [41]. Quality of Life: McManus et al. [43]. Anxiety Scale: Hickey et al. [41]. Technology-based Assessment and ECG monitoring (Holter or Mobile device): Halcox et al. [42]. Finally, patient health medical records (demographics, medical history, health behaviours): Turakhia et al. [44].

The study that utilised the majority of assessments was that undertaken by Hickey and Freedson [41]. Hickey and Freedson [41] used 10 instruments (n = 10) in conjunction with baseline and monthly data recording (via electronic medical records system review) throughout the six-month duration of the study. The 10 assessments deployed were: the Atrial Fibrillation Knowledge Scale (AFKS) [46]; the Canadian Cardiovascular Society Severity in Atrial Fibrillation scale (CCS-SAF) [47]; the Atrial Fibrillation Effect on Quality of Life (AFEQT) [48]; the Control Attitudes Scale-Revised (CAS-R) [49]; the Morisky 4-item Self-Report Measure of Medication-Taking Behaviour (MMAS-4) [50,51]; the Self-Efficacy for Appropriate Medication Use Scale (SEAMS) [52]; the Short Form Health Survey (SF-36 Quality of Life) [53,54]; European Questionnaire 5 Dimensions (EQ-5D) [55,56]; the Patient Health Questionnaire (PHQ-9) [57]; and the State Trait Anxiety Inventory (STAI) [58].

The study design theme encompasses three secondary themes: duration, study criteria, and study type. Across the studies (depending upon the study design), some studies required their patients/participants to provided additional information in conjunction to their respective mobile ECG reading. Readings were taken over various times in the respective studies. Halcox et al. [42] measured twice per week for 12 months, while Hickey and Freedson [41] recorded three times per week over a six-month period. Aronsson et al. [37], Evans et al. [40], Turakhia et al. [44], used a two-week period. Doliwa, Rosenqvist, and Frykman [38] patients used the Zenicor ECG device over a 30-day period while McManus et al. [43] used a 2-min waveform reading. Five studies stated that their study design included study criteria (i.e., inclusion/exclusion). For example, Doliwa, Rosenqvist, and Frykman [38] recruited 22-participants with a diagnosis of symptomatic paroxysmal AF. While Haberman et al. [26] recruited 335 participants from Division I athletes, healthy young adults, and cardiology clinics. Hickey and Freedson [41] recruited adults >18 years, who had a 30-day history of AF, were either male or female, able to use a smartphone, and participants who were able to read and receive text messages on the day of enrolment onto the study. Furthermore, Turakhia et al. [44] recruited participants from cardiology, echocardiography and stress-testing clinics with additional inclusion criteria of specific age and having a minimum of two risk factors. The exclusion criteria included prior AF diagnoses, stroke, transient ischemic attacks, implantable pacemaker or defibrillator or someone who experienced palpitations or syncope in the previous year [44]. The McManus et al. [43] study was the one investigation aimed at testing a hypothesis using a prototype which measured waveforms via the iPhone 4S. Across all selected studies, each one reported a different study type (i.e., RCT or comparative). None of the nine studies reported the same study design.

## 4. Discussion

### Principle Findings

This review paper provides a contemporary insight into the growing field of mobile ECG monitoring and detecting AF that coincides with palpitations. Out of 981 abstracts, a total of nine papers were selected for a comprehensive examination. A total of six primary themes were identified and, within each primary theme, a series of secondary themes were ascertained. Given the increasing use of wearable devices coupled with the increase in ageing populations and the drive to provide cost-effective primary care, the evidence lends itself to the adoption of mobile ECG monitoring into care practice and policy. As stated earlier in this paper, NICE [10] in the UK are primarily using the AliveCor Kardia ECG device.

The primary themes, environment and population, highlight the range of research centres, laboratories, and geographic locations that have investigated ECG mobile devices. Nonetheless, there is an argument that these selected studies are community based. However, the participants are not reported to have been recruited through a physician, surgery, or from a hospital via a cardiologist. The theme of population highlighted the varying sample sizes and how, in some instances, participants were recruited through cardiology clinics or departments; nevertheless, this was limited to three studies. Overall, the age range of participants illustrated a spread across populations; ensuring patients of all ages who presented with palpitations were involved in the studies. The primary theme wearable devices encompassed commercial, wearable, and purpose-built technology as a means of detecting and measuring AR and AF during periods when palpitations are prevalent. Overall, four studies used the AliveCor Kardia device, accessible via the Apple iPhone, while two studies deployed the commercial ECG device Zenicor. To date, there have only been a handful of studies published in academic journals [13,14,15], or via the Zenicor website [59] that examine purpose-built or commercial ECG devices.

With the exception of Evans et al. [40], no other selected study provided an insight into the use of mobile ECG devices and monitoring from the perspective of health practitioners. The final nine selected papers provide insights into the varying rationales for monitoring palpitations and AR across populations. We suggest that undertaking a community-based approach that included a physician (s) or consultant cardiologist would offer greater insights into the benefits of mobile ECG monitoring from the viewpoint of both health practitioner and providers.

All nine studies used an assortment of assessments and measures, which formed 11 secondary themes. The assessment theme indicates the complexity of deploying mobile ECG devices in conjunction with additional health outcomes to ascertain patient’s levels of anxiety, quality of life, and the detection of AF through self-reporting and/or clinical practitioners. Varying study designs were executed. Nonetheless, given the limited duration of assessment, the results showed a positive trend in detecting AF. While AF and AR may vary across populations, the studies did report that patient response via the technology occurred at the time of the patient experiencing palpitations. Furthermore, the objectives of the studies also varied and ranged from validation to feasibility, cost effectiveness and clinical trials. This is further evidenced by the increase in clinical trials, as noted in this review’s introduction. Consequently, the many clinical trials at various stages across the world highlight the widespread interest in this application of mobile health technology. However, drawing comparisons across the selected studies is problematic based upon the varying environments, assessments, populations, and wearable devices. While the cost effectiveness is a principal concern for primary care and health care strategists, preliminary evidence from this review (Table 2), coupled with findings from recent international studies such as Hendrikx et al. [13], Usadel et al. [14] and Dahlqvist et al. [15], demonstrates the great potential of deploying wearable ECG devices.

## 5. Limitations

One of the limitations of this scoping review is that each database requires its own set of limiters. Consequently, each database search is slightly different as demonstrated in Table 3. The database searches did not identify the papers of Hendrikx et al. [13], Usadel et al. [14] and Dahlqvist et al. [15] that are available via the Zenicor website [59]. These papers were published in April and May 2018 and therefore outside of the time period of this scoping literature review. Furthermore, these papers did not fit the inclusion criteria because their primary area of investigation centred on the diagnosis and treatment of stroke patients. Although the Dahlqvist et al. [15] paper fits the inclusion criteria, the authors decided not to include it in Table 2 given that it did not appear in the search period. Moreover, the Dahlqvist et al. [13] study did not fit the >18 age inclusion criteria of this scoping review as participants in the respective study were children aged between 5 and 17 years old. Another paper was excluded because it was published in Swedish [60] and consequently failed the English language inclusion criterion.

## 6. Future Research

Based on the findings of this review, the authors propose several areas for furthering and expanding this research:

1. Future work may wish to consider undertaking a systematic review in order to synthesise existing and recently published work. This systematic review could include development features, accuracy, algorithms, utility and reproducibility, in addition to diagnostics and user/patient experience(s).
2. Following the work of Evans et al. [40], clinicians and researchers alike should consider exploring the use of mobile ECG devices from the standpoint of health practitioners working in the delivery of primary and community care.
3. Implementing and conducting qualitative data collection in future studies would provide a greater insight and understanding of the needs, apprehensions, and expectations of patients and primary care practitioners. Simultaneously, this would provide the opportunity to examine the role of patient’s and support networks. Evans et al. [40] illustrated the potential opportunities for mobile ECG monitoring in low, middle income countries (LMICs), and by their approach has the potential to offer substantial changes in developed and developing regions.
4. Future investigations should explore the adherence and adoption of mobile ECG devices, learning from previous health, gerontological and ICT studies [61,62,63,64,65,66,67,68]. Existing research in different fields has demonstrated how technology has been used and evaluated by community dwelling adults living in different geographic locations. Understanding people’s motivations and behaviour in relation to technology would significantly support future work in this field. In addition, the impact of technology efficacy by health practitioners on service delivery could be assessed.
  - Privacy and security issues and concerns surrounding data need to be addressed from a multi-disciplinary standpoint. Further work is needed to explore the use of wearable devices from a clinical environment and conducting qualitative data to gain an in-depth insight into the concerns of patients, support networks and practitioners.
5. Future studies should determine the exact cost effectiveness of deploying mobile ECG devices with the aim of providing evidence to health care strategists, governments and managers of the benefits of this form of technology in the community. Such studies could have a significant impact in the care pathways following the detection of AR and AF.
6. To ascertain how mobile ECG devices could affect the delivery of primary care, we suggest that a large-scale feasibility study, encompassing variable populations (i.e., age range, ethnicity and socio economic), should be conducted to provide results to different actors (i.e., government, health care practitioners, health care strategists, researchers, patients and support networks). It is important that such studies include as full a range of actors as possible from primary care physicians, cardiologists, patients, lay people, patients’ support networks, health organizations (i.e., NICE), and government funding agencies.
7. Future scoping reviews should follow the recent extension to the existing PRISMA protocol—the PRISMA Extension for Scoping Reviews (PRISMA-ScR) [68].

The findings of this scoping review contribute to the fields of primary care, medicine and wearable devices. Nonetheless, ECG wearable devices are directly available to consumers via retail outlets and online websites. With this in mind, there is a risk to users who choose to purchase devices from online stores or directly from suppliers or manufacturer’s website. Users may not fully understand the recordings or misunderstand the information presented to them. For example, the accurate interpretation of output statistics and the recognition of any false positives and false negatives. Thus, this leads to a myriad of issues for clinicians, users, and carers and may have health consequences for individuals and cost implications for service providers. These devices while readily available on the market have not necessarily gone through the process of being categorised as medical devices. This issue was raised by Marston and Smith [65,66] concerning the delivery of physiotherapy via videogame consoles. Consequently, Marston and Smith [65,66] argued for the need and requirement of videogame consoles to undergo some form of official categorization and approval rating. While there are worldwide videogame classifications [66], this is not the case for wearable devices and in particular mobile ECG devices. We argue for a requirement for the manufacturers of mobile ECG devices to gain FDA and European Medicines Agency (EMA) approval. However, as noted by Mantovani and Bocos [67] and Wiersinga [69], gaining FDA and EMA approval is not a straightforward process and, given the phenomenal developments within this domain, applying for medical device classification could be very time consuming. However, Wiersinga [69] discusses in depth the regulation processes for medical devices from the standpoint of industry and proposed recommendations for best practice.

## 7. Conclusions

This review is distinctive because it demonstrates positive trends to using and deploying mobile ECG devices across different environmental settings and populations. With global populations set to increase over the coming decades, the need to identify alternative solutions to facilitate and ensure primary care providers are able to deliver cost-effective health care is crucial, for both the service provider and the patient [9]. Detecting and diagnosing AR/AF using mobile ECG devices would reduce the risk of morbidity and associated health implications such as stroke or mortality [5,6,7,70].

Based on the evidence displayed in this review, the authors believe substantial work is warranted at both a national and international scale with a view to supporting primary care providers to deliver high levels of care at a low cost to the service provider. This, in turn will alleviate patient anxiety, risk of morbidity and mortality. In addition, it would positively impact on national and international guidelines concerning pathways to care.

## Figures and Tables

**Table 1:** Criteria for study selection.

Inclusion	Exclusion
Mobile apps (mApps)	Master’s and PhD thesis
Electrocardiogram (ECG/EKG)	Conference proceedings
Cardiogram	Book Chapters
Wearables	Reports
Atrial Fibrillation (AF)	Reviews
Heart	Pulse monitoring
Human	Theoretical papers
ECG Wearable Devices/Patches	Athletes
Mobile Health (mHealth)	Defibrillators
Security/Privacy	Intensive care unit (ICU) or high dependency unit (HDU)
Smart Fabric/textiles	WSBN
Papers published in Journals	Animals/non-human
Commercial technologies	Co-morbidities (i.e., transplant patients)
Purpose-built technologies	Newsletters
Encryption	Editorials
Big Data	PhD, MSc & BSc Thesis
Human	
Study designs: (randomised control trial (RCT), Exploratory, Cohort, Prospective, Feasibility)	

**Table 2:** Databases searched, search terms used, and adaptations employed.

Database	Search Term Used	Adaptions
Association for Computing Machinery (ACM)	(Arrhythmia Atrial Fibrillation ECG EKG Palpitations wearables) AND (-Algorithms -map -sensor -consumer -mathematical -statistical) AND keywords. author. keyword:(Arrhythmia Atrial Fibrillation ECG EKG Palpitations wearables -wavelet -brain -skin -posture -music -grasp -grip -sonic -speculative) AND record Abstract: (Arrhythmia Atrial Fibrillation ECG EKG Palpitations wearables)	Manufacturers’/generic names not recognised. NOT any: Algorithms map sensor consumer mathematical statistical. Keyword NOT: wavelet brain skin posture music grasp grip sonic
CINHAL	(TX (“Palpitations” OR “Arrhythmia” OR “Atrial Fibrillation”)) AND (TX “Wearable ECG”) AND (TX “Wearable EKG”) OR (TX (“Wearable technologies” OR Wearable devices)) NOT (TX (“Catheter” OR “Surgery” OR “Ablation” OR “Catheter ablation” OR “Nursing Practice” OR “Gait”)) NOT (TX “Students”)	Manufacturers’/generic names not recognised. NOT “Catheter” OR “Surgery” OR “Ablation” OR “Catheter ablation” OR “Nursing Practice” NOT “Students”
Google scholar (wearable device)	ECG EKG Alive OR Cor OR Zoe OR Patch OR Scanadu OR Scout OR Perminova OR CoVa OR necklace OR Kardia OR ECG OR Necklace OR Cardio OR Analytics OR Heal OR Force OR Smart OR Cardio OR Beurer OR ME80 OR Beurer OR PM2 “wearable device”	Excluded patents
Google scholar (wearable technology)	ECG EKG Alive OR Cor OR Zoe OR Patch OR Scanadu OR Scout OR Perminova OR CoVa OR necklace OR Kardia OR ECG OR Necklace OR Cardio OR Analytics OR Heal OR Force OR Smart OR Cardio OR Beurer OR ME80 OR Beurer OR PM2 “wearable technology”	Excluded patents
PubMed	Palpitations OR Arrhythmia OR Atrial Fibrillation And (ECG) AND (EKG) OR Wearable technologies OR Wearable devices)) AND (Alive Cor OR Zoe Patch OR Scanadu Scout OR Perminova CoVa necklace OR Kardia OR ECG Necklace OR Cardio Analytics OR Heal Force OR Smart Cardio OR Beurer ME80 OR Beurer PM25 OR Prince 180B OR Cardea SOLO OR Spyder Pro OR Spyder Personal OR MiCor A100)) NOT (sport AND algorithms))	AND NOT sport AND algorithms
PubMed MESH	Wearable devices OR Wearable technologies AND (ECG OR EKG) AND (Palpitations OR Arrhythmia OR Atrial Fibrillation)	Manufacturers’/generic names not recognised. AND NOT sport AND algorithms
Scopus	Palpitations OR Arrhythmia OR Atrial Fibrillation And {ECG} AND {EKG} OR Wearable* AND techonolo* OR device AND NOT algorithms	Manufacturers’/generic names not used Use wildcard* AND NOT algorithms
Scopus	Alive Cor” OR “Zoe Patch” OR “Scanadu Scout” OR “Perminova CoVa Necklace” OR “QardioCore” OR “Kardia” OR “ECG Necklace” OR “Cardio Analytics” OR “Heal Force” OR “Smart Cardio” Or “ChoiceMMed” OR “Beurer ME80” OR “Beurer PM25” OR “Zodore” OR “Prince 180B” OR “Cardea SOLO” OR “Spyder Pro” OR “Spyder Personal” OR “MiCor A100”)	Dropped: “Palpitations” OR “Arrhythmia” OR “Atrial Fibrillation” AND “ECG” OR “EKG”

**Table 3:** Summary of articles (N = 9) included for this scoping review.

1st Author Year Country	Objectives	Participants	Study Design	Assessment(s)	Technology	Main Findings
Aronsson et al. [40] 2015 Sweden	To estimate the cost effectiveness of 2 weeks of intermittent screening for asymptomatic atrial fibrillation (AF) in 75/76-year-old individuals.	n = 25,415 Aged 75–76 years Female 55.9%	Observational Cohort study	In total, 30-s recordings taken twice daily, or when symptoms of palpitations for 2 weeks.	Zenicor EKG device	With the use of a decision analytic simulation model, it has been shown that screening for asymptomatic AF in 75/76-year-old individuals is cost effective.
Doliwa, Rosenqvist, and Frykman [41] 2012 Sweden	To compare short intermittent heart rhythm recording with or without symptoms with continuous ECG recordings for 30 days, with two registrations of 10 s per day.	n = 22 Aged 46–77 years Females 27% Median age 63 years	Experimental study, randomised controlled blinded trial	Recordings were taken twice daily; once in the morning and once in the evening for a 30-day period. Participants were asked to record when experiencing arrhythmia symptoms (recorded as symptomatic).	Zenicor EKG device	AF episodes were diagnosed in 18 (82%) patients compared with seven (32%) patients using continuous ECG, (*p* = 0.001. Short-term ECG registrations over extended periods of time seem to be a more sensitive tool, compared with short continuous ECG recordings, for the detection of AF episodes.
Boudreau et al. [42] 2017 USA	To determine the validity of eight monitors for Heart Rate (HR) compared with an ECG and seven monitors for Energy Expenditure (EE) compared with a metabolic analyser during graded cycling and resistance exercise.	n = 50 Aged 18–35 years Female n = 28 (56%) Mean age 22.71 ± 2.99	Experimental comparative study	Session 1: Performed a graded exercise test on a cycle ergometer. Session 2: Performed a graded exercise test of four different strength training exercises on a resistance exercise machine. Repeated 3 days later in the laboratory. Exclusion: Cardiovascular disease or musculoskeletal injury in the last 6 months.	Apple Watch Series 2, Fitbit Blaze, Fitbit Charge 2, Polar H7, Polar A360, Garmin Vivosmart HR, TomTom Touch, and Bose SoundSport Pulse (BSP) headphones	This study revealed that both HR and EE differed among the eight wearable devices during both cycling and resistance exercise and had varying levels of validity when compared with a six-lead ECG and metabolic analyser. It was also observed that HR measures from wearable devices were more accurate at rest and lower exercise intensities than at higher intensities. Among tested devices, HR accuracy, as reflected by intraclass correlation and MAPE values, was highest in the PH7, BSP, and AWS2. The PH7 and AWS2 also proved to provide more accurate caloric estimations than other devices. HR from wearable devices differed at different exercise intensities; EE estimates from wearable devices were inaccurate.
Evans et al. [43] 2017 Kenya	To examine the feasibility of using mobile ECG recording technology to detect AF.	n = 50 Mean age 54.3 ± 20.5. Females 66%	Prospective observational study	Of 2-week duration. In a rural community. Health practitioners (physicians, clinical officers, nurse) completed a self-assessment of a 4-item scale relating to ICT access, knowledge/interpretation of results and perception of AF in the community.	AliveCor Kardia Mobile ECG device	ECG tracings of four of the 50 patients who completed the study showed AF (8% AF yield), and none had been previously diagnosed with AF. Using mobile ECG technology in screening for AF in low-resource settings is feasible and can detect a significant proportion of AF cases that will otherwise go undiagnosed. Further study is needed to examine the cost effectiveness of this approach for the detection of AF and its effect on reducing the risk of stroke in developing countries.
Haberman et al. [26] 2015 USA	Compare the standard 12-lead ECG to the smartphone ECG in healthy young adults, elite athletes, and cardiology clinic patients. Accuracy for determining baseline ECG intervals and rate and rhythm was assessed.	n = 335 Mean age 35 ± 20 Female 51%	Experimental comparative study	Using an iPhone case or iPad, 30-s lead iECG waveforms were obtained. Standard 12-lead ECGs were acquired immediately after the smartphone tracing was obtained. De-identified ECGs were interpreted by automated algorithms and adjudicated by two board-certified electrophysiologists	AliveCor device (30-s ECG wireless reading). Patients trained over 1–2 min to take their own readings	This study provides evidence that wireless ECG devices can be used on a large scale to detect rate, conduction intervals and AF. Incorporation of automated discrimination, with enhanced smartphone features with notification capability and decision support. Both smartphone and standard ECGs detected atrial rate and rhythm, AV block, and QRS delay with equal accuracy. Sensitivities ranged from 72% (QRS delay) to 94% (atrial fibrillation). Specificities were all above 94% for both modalities.
Hickey et al. [44] 2016 USA	The primary aims of the iHEART study are to: (1) document AF using real-time ECG capture; (2) evaluate the impact on AF treatment and Quality-Adjusted Life Years (QALYs); and (3) evaluate the effectiveness of text messaging on AF knowledge and promoting proactive self-management of multiple chronic conditions	n = 300 Aged > 18 years	Study protocol, observational study. Single-centre prospective	ECG reading taken at baseline. Complete all questionnaires at baseline and at 6 months. Questionnaires included the Atrial Fibrillation Knowledge Scale, the Canadian Cardiovascular Society Severity in Atrial Fibrillation scale, the Atrial Fibrillation Effect on Quality of Life, the Control Attitudes Scale-Revised, the Morisky 4-item Self-Report Measure of Medication-Taking Behaviour, the Self-Efficacy for Appropriate Medication Use Scale, the Short Form Health Survey, European Questionnaire 5 Dimensions, the Patient Health Questionnaire, and the State Trait Anxiety Inventory.	iPhone, AliveCor Mobile ECG Kardia app	This will be the first study to investigate the utility of a mobile health intervention in a “real world” setting. We will evaluate the ability of the iHEART intervention to improve the detection and treatment of recurrent atrial fibrillation and assess the intervention’s impact on improving clinical outcomes, quality of life, quality-adjusted life-years and disease-specific knowledge.
Halcox et al. [45] 2017 UK		n = 1001, Mean age 72.6 ± 5.4 Females 53.34%	Experimental study	Baseline characteristics. Participant experience survey (completed at the end of the study). Questions included anxiety about their heart rhythm problems, more likely to visit their doctor, or prefer to switch to a study group (responses reported via a 10-point visual analogue scale). iECG patients were asked about ease of use, restriction of activities, anxiety, concern about data security and a general satisfaction with the device (via 5-point Likert scale). Health economics were estimated from the UK National Health Service (NHS) and personal social services, using data from the study activity and relevant costs.	AliveCor Kardia device	Screening with twice-weekly single-lead iECG with remote interpretation in ambulatory patients ≥65 years of age at increased risk of stroke is significantly more likely to identify incident AF than RC over a 12-month period. This approach is also highly acceptable to this group of patients, supporting further evaluation in an appropriately powered, event-driven clinical trial.
McManus et al. [46] 2016 USA	To test whether an enhanced smartphone app for AF detection can discriminate between sinus rhythm (SR), AF, premature atrial contractions (PACs), and premature ventricular contractions (PVCs).	**AF—**n = 98 65.9 ± 12.2 Male—n = 70 (71.4%) White n = 91(92.9) **PAC—**n = 15 73.1 ± 5.9 Male—n = 11 (73.3%) White n = 14(93.3) **PVC—**n = 15 62.8 ± 13.8 Male—n = 9 (60%) White n = 13(86.7) **Sinus Rhythm—**n = 91 66 ± 11.9 Male—n = 63 (69.2%) White n = 86(94.5)	Experimental study	Analysis of 219 2-min pulse recordings. Usability questionnaire to sub-group of ns = 65 app users. Examined the sensitivity, specificity, and predictive accuracy of the app for AF, PAC, and PVC discrimination from sinus rhythm using the 12-lead EKG or 3-lead telemetry as the gold standard.	PULSE-SMART prototype App used via the iPhone 4S	The smartphone-based app demonstrated excellent sensitivity (0.970), specificity (0.935), and accuracy (0.951) for real-time identification of an irregular pulse during AF. The app also showed good accuracy for PAC (0.955) and PVC discrimination (0.960). The vast majority of surveyed app users (83%) reported that it was “useful” and “not complex” to use.
Turakhia et al. 2015a [47] USA	To detect silent AF in asymptomatic patients with known risk factors through screening for AF using continuous ambulatory ECG.	n = 75, Mean age 69 ± 8.0 years. Male only	Observational study, single centre	Records up to 14 days of monitoring on a single vector. Participants press the symptomatic trigger on the device if symptoms presented. Patient diary, detailing symptoms. Baseline characteristics: demographics, medical history, ECG parameters, health behaviours were abstract from patient medical record by two trained investigators.	Zio wearable patch-based device	AF was detected in four subjects (5.3%; AF burden 28–48%). Atrial tachycardia (AT) was present in 67% (≥4 beats), 44% (≥8 beats), and 6.7% (≥60 s) of subjects. The combined diagnostic yield of sustained AT/AF was 11%. In subjects without sustained AT/AF, 11 (16%) had ≥30 supraventricular ectopic complexes per hour Outpatient extended ECG screening for asymptomatic AF is feasible, with AF identified in one in 20 subjects and sustained AT/AF identified in one in nine subjects, respectively. We also found a high prevalence of asymptomatic AT and frequent supraventricular ectopic complexes, which may be relevant to development of AF or stroke.

## Abbreviations

ACM	Association of Computing Machinery
AF	Atrial Fibrillation
AFEQT	Atrial Fibrillation Effect on Quality of Life
AFKS	Atrial Fibrillation Knowledge Scale
AR	Arrhythmia
CAS-R	Control Attitudes Scale-Revised
CCS-SAF	Canadian Cardiovascular Society Severity in Atrial Fibrillation scale
CE	Cost Effectiveness
ECG/EKG	Electrocardiogram
EE	Energy Expenditure
EQ-5D	European Questionnaire 5 Dimensions
HR	Heart rate
mApp	Mobile Apps
NHS	National Health Service
NICE	National Institute for Health Care Excellence
PHQ-9	Patient Health Questionnaire
PRISMA	Preferred Reporting Items for Systematic Reviews and Meta-Analyses
QALY	Quality-Adjusted Life Years
QOL	Quality of Life
RCT	Randomised controlled trial
SEAMS	Self-Efficacy for Appropriate Medication Use Scale
STAI	State Trait Anxiety Inventory
UK	United Kingdom
USA	United States of America
VA	Veteran Affairs
